# Investigation of the Connection Schemes between Decks in 3D NAND Flash

**DOI:** 10.3390/mi14091779

**Published:** 2023-09-17

**Authors:** Jianquan Jia, Lei Jin, Kaikai You, Anyi Zhu

**Affiliations:** 1 Institute of Microelectronics of the Chinese Academy of Sciences, Beijing 100029, China; 2 University of Chinese Academy of Sciences, Beijing 100049, China

**Keywords:** 3D NAND, dual-deck, connection scheme, poly-plug, device reliability

## Abstract

Dual-deck stacking technology is an effective solution for solving the contradiction between the demand for increasing storage layers and the challenge of the deep hole etching process in 3D NAND flash. The connection scheme between decks is a key technology for the dual-deck structure. It has become one of the necessary techniques for 3D NAND flash storage density improvement. This article mainly studies the impact of connection schemes between decks on cell reliability. Based on experimental data and simulation analysis, unfavorable effects were found as the gate channeling the breakdown and data retention characteristics of the top cells in the lower deck deteriorated due to the local electric field enhancement in the connection scheme without a poly-plug. This mainly contributed to the structural change of these cells within process impact. They will suffer secondary etching during the upper deck channel etching process due to alignment issues between the upper and lower decks. In another scheme with a poly-plug connection between decks, the saturation current of the channel decreased and the current variation increased. The fundamental cause of the current anomaly is that the Poly-plug has a certain shielding effect on channel inversion and the weak inversion region becomes a bottleneck for the channel current. The increase in variation is due to the shielding effect differences in the different structures of the poly-plug. Therefore, for the connection scheme without a poly-plug, the article proposes to improve device reliability by increasing the oxide thickness between decks and setting the top cells of the lower decks to be virtual cells. For the connection scheme with a poly-plug, the plug‘s N-type doping scheme is proposed to avoid the current dropping anomaly.

## 1. Introduction

**Figure 1:**
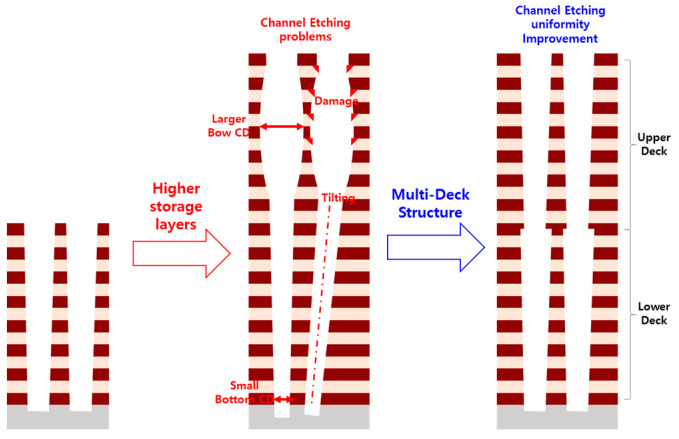
The dual-deck technology is crucial for addressing deep hole etching challenges as the number of storage layers increases in 3D NAND flash.

3D NAND flash memory is widespread used in various applications to fulfill the explosive growth of data demand due to good product performance and cost [1]. Increasing the number of storage layers is one of the main methods to improve the storage density of 3D NAND flash [2]. As the number of memory layers increases, the relatively large aspect ratio of etching channel holes becomes a severe challenge in single-deck 3D NAND flash [3]. The application of multi-layer stacking technology can effectively reduce the pressure of the deep hole etching process [4,5,6]. It has important significance for improving the density of 3D NAND flash memory. As shown in Figure 1 below, as the number of storage layers continues increasing, the etching depth of the channel gradually increases. However, deep hole etching with a large aspect ratio is difficult to achieve in the process. There are some etching problems with the storage layers increasing, such as channel etching tilting and small diameter of bottom cell, seriously affecting device reliability. Adopting the multi-stack structure, the etching process could be divided into multiple steps in channel etching. The etching depth significantly reduced by step, effectively ensuring the uniformity of the cell structure, which is helpful for cell reliability. This solution is of great milestone-worthy significance for the development of 3D NAND flash ultra-high-layer products. However, it needs to be noted that the multi-deck technology will introduce some new reliability problems, such as the program disturbance issue in the dual-deck structure reported by X.J et al. [7]. It is also crucial to check whether the connection schemes would cause new reliability problems in 3D NAND flash.

This article mainly analyzes the impact of two kinds of mainstream connection schemes between decks on cell reliability in dual-deck 3D NAND flash. In the connection scheme without a poly-plug, due to secondary etching caused by the alignment problem of the upper and lower decks, the structure of the top cells in the lower deck will be altered, resulting in poorer retention and WL to CH breakdown. It proposes increasing the oxide thickness between the upper and lower decks and setting some top cells as virtual cells to slow down this problem. In another scheme with a poly-plug connection, the saturation current of the channel decreases while the structure variation increases. The poly-plug becomes a bottleneck for the channel current due to its local shielding effect. Thus, the article proposes the use of a poly-plug N-type doping scheme, and experiments show that this can effectively solve the current abnormality problem.

## 2. Methods and Structures

This article’s research was carried out through a combination of experiment and simulation, which is also one of the main research methods in the field of 3D NAND flash [8,9,10]. Simulation was used to analyze physical mechanisms and experimental testing was used for verification. For the experimental part, a desktop level FPGA (field programmable gate array) and Wafer level tester were used to measure the basic electrical characteristics of the device. The models used in TCAD device simulation were as follows: the Shockly–Read–Hall (SRH) model, the non-local tunneling (NLT) model, the Poole–Frenkel model, the thermal emission model, and the drift-diffusion model. These models can effectively reflect physical characteristics and have proven to be useful in explaining many phenomena of 3D NAND flash [11,12], and the specific simulation parameters used can be referenced in article [13], which is a previous research study by the research group.

**Figure 2:**
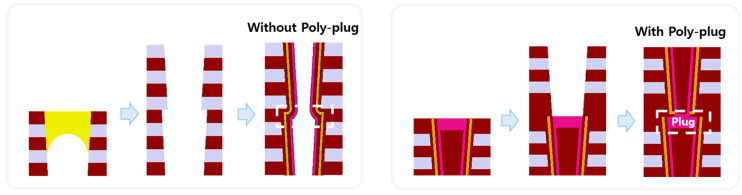
The 3D Dual-Deck NAND flash process flows and structures with/without poly-plug connection schemes.

The two main connection schemes studied in 3D dual-deck NAND flash in this article are as follows. As shown on the left side of Figure 2, the first scheme is a connection scheme without poly-plug. It mainly uses the carbon plug fill-and-remove scheme to protect the lower deck during the channel hole etching of the upper deck and subsequently forms the final structure through the upper deck and lower deck gate stacks and channel poly one-time deposition [14]. As shown on the right side of Figure 2, the other scheme is a connection scheme with a poly-plug. It mainly uses a poly-plug as the channel conductive connection between the upper and lower decks, so the deposition of gate stack and channel polysilicon between the upper and lower decks divides into two steps.

## 3. Experiments and Simulations

**Figure 3:**
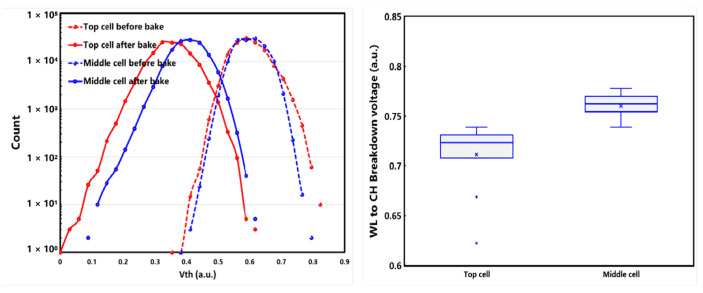
Device data retention and breakdown resistance characteristics of top cells in lower deck in 3D dual-deck NAND without a poly-plug.

Some abnormal device characteristics were observed in the experiment in the 3D dual-deck NAND flash structure without a poly-plug connection. As shown on the left side of Figure 3, the experimental data shows that the device retention characteristics of the top cell in the lower deck deteriorated compared to the other cells. At the same time, as shown on the right side of Figure 3, these cells’ breakdown voltage between the gate and channel also decreased. The deterioration of these device characteristics seriously affects the reliability of 3D NAND flash memory chips.

**Figure 4:**
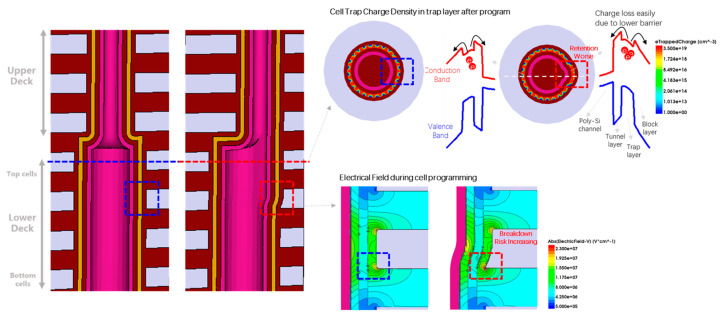
Simulation analysis of characteristics degradation for the top cells in lower deck in 3D dual-deck NAND without a poly-plug.

Combining process flow and simulation analysis, as shown in Figure 4, the device’s characteristics degradation of the top cells in the lower deck mainly comes from the connection scheme between the decks. When performing the channel hole etching process of the upper deck, there is a need for channel hole alignment between the upper and lower decks. However, due to non-ideal effects such as stress, there may be overlap mismatch of the decks, which causes a secondary etching for the top cells of the lower deck, resulting in changes to the cell structure. According to the simulation diagram from Figure 4, after the device structure is altered, there will be local trap charge accumulation in the trap layer, due to the strong local electric field effect during programming. Due to a lower barrier, this leads to faster electron loss in this part of the device during subsequent data retention, resulting in poor data retention reliability [15]. At the same time, some abnormal structures may experience a peak electric field enhancement effect, as shown in the simulation diagram at the bottom right of Figure 4, causing a decrease in the breakdown between the gate and the channel.

**Figure 5:**
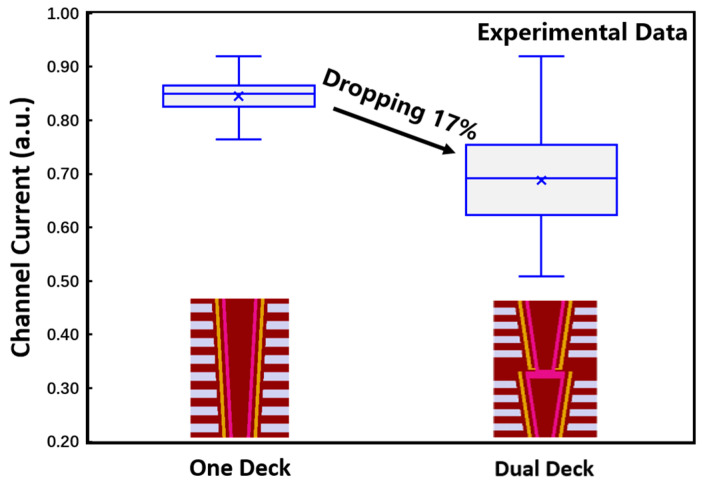
The difference in the channel saturation current between one-deck and dual-deck 3D NAND flash based on experimental data.

For investigation of the connection scheme with a poly-plug, the channel saturation currents were measured on samples with a one-deck structure and a dual-deck structure, respectively. For contrast, the number of storage layers in all the samples were the same. As shown in Figure 5, the phenomenon of current falling and large variation was observed in the dual-deck samples. The typical value of the current dropped by 17% and the variation in the current almost doubled. This will affect the uniformity of array cells and reduce the reliability of the NAND flash.

**Figure 6:**
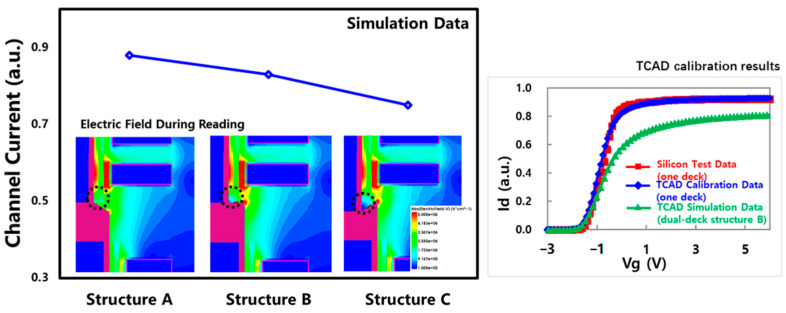
Simulation analysis of the channel saturation current anomalies in dual-deck 3D NAND with a poly-plug connection scheme.

A basic dual-deck 3D NAND structure with the poly-plug connection scheme was set up by TCAD, labeled structure B in Figure 6. The simulation was first calibrated based on the experimental test data of a one-deck structure. The simulation further shows that the channel saturation current of the dual-deck structure with a poly-plug connection scheme was lower than the one deck structure, as shown on the right side of Figure 6. During the simulation of the reading operation, it was found that the electric field at the corners of the connection (black dashed circles) was weak due to the shielding effect of the poly-plug on nearby WLs, which become a bottleneck for the channel saturation current. Due to the chemical–mechanical polishing (CMP) process used to flatten the structure of the lower deck before depositing the upper deck, there are some differences in the structure of the poly-plug under process variation, such as structure A and structure C shown in Figure 6. Their electric field shielding effect on nearby WLs is different in the simulation. Thus, the structural difference of a poly-plug is the main reason for variations in channel saturation current.

In summary, this chapter mainly focuses on simulation analysis of abnormal device characteristics displayed in experimental testing of dual-deck 3D NAND flash with different connection schemes between decks. In the scheme with a poly-plug connection, the data retention characteristics and breakdown voltage of the top cells in the lower deck reduced, due to the alignment problem between the upper and lower decks. This is mainly because these cells’ structures changed due to suffering secondary etching, making it easier to generate local electric field enhancement effects. In another common dual-deck scheme with a poly-plug connection, the poly-plug would generate an electric field shielding effect on the nearby WLs, becoming the bottleneck for channel saturation current, leading to an abnormal decrease in channel saturation current. Due to differences in the structures of the poly-plug, the variation of channel saturation current would also increase.

## 4. Proposals and Results

**Figure 7:**
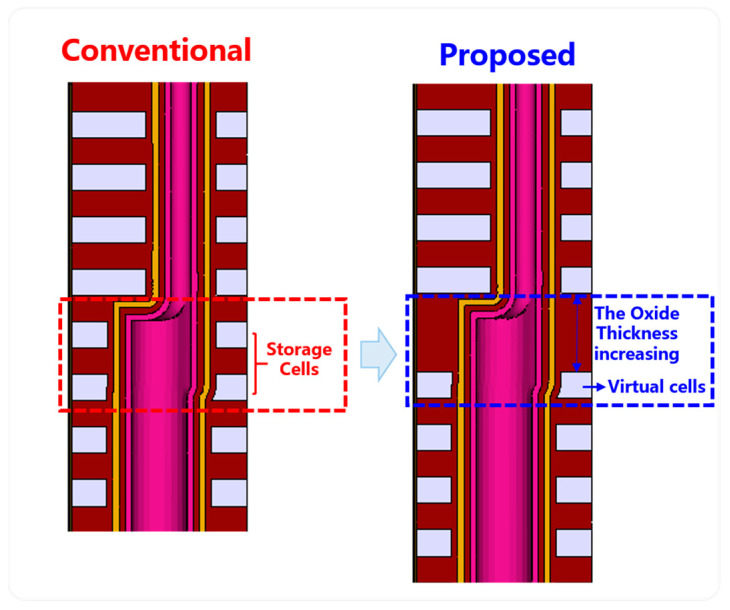
The proposal scheme diagram for 3D dual-deck NAND without a poly-plug connection.

Based on the analysis in the previous part of the article, it proposes to increase the thickness of the oxide between decks for the connection without a poly-plug scheme. At the same time, the method of setting the top cells in the lower deck as the virtual cells also proposes to reduce the risk of device characteristic degradation caused by the presence of overlap mismatch. As shown in Figure 7, increasing the oxide thickness between decks can buffer the etching window and reduce damage to the storage cells in the lower deck. Meanwhile, the top cells of the lower deck are set as virtual cells, which can effectively ensure the reliability of the entire chip’s data storage. Although a certain amount of storage capacity may be sacrificed, the proportion of capacity loss is negligible compared with the rapid increase in stack capacity of the upper decks.

**Figure 8:**
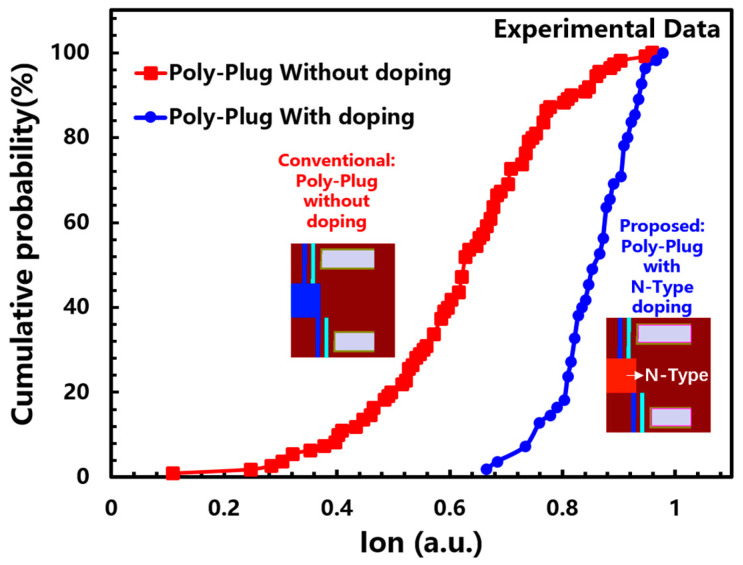
The experimental validation data of the proposed scheme for 3D dual-deck NAND with a poly-plug connection.

In the scheme with a poly-plug connection, this article proposes the poly-plug N-type doping scheme to improve channel current and solve the variation problem. The proposal scheme is based on the principle that channel current relying on the inversion and the structure of the plug has an electric field shielding effect on the channel inversion. Through the poly-plug N-type doping scheme, the channel current conduction at the corners of the poly-plug relies on impurity electrons rather than inversion electrons, which can avoid the disadvantage of a poly-plug as a current bottleneck. The experiment verified this point, as shown in Figure 8, and the channel saturation current increased significantly after poly-plug N-type doping, while its variation improved significantly.

## 5. Discussion

This chapter mainly compares two connection schemes between decks in 3D dual-deck NAND flash, as shown in Table 1 below. First, comparison from the process and cost, in the connection scheme with a poly-plug, the gate stacking and channel polysilicon in the lower deck and upper deck require a two-step deposition, which causes a relatively complex process and high cost. Second, the impact of these two schemes on device reliability is also different. The former mainly affects the characteristics of some cells, while the latter mainly affects the uniformity of the entire storage array. Finally, comparing the negative effects of the proposed schemes in the article, the article proposes a scheme to increase the oxide thickness and set virtual cells for the former, which will cause storage capacity loss, but the proportion of loss is relatively small. As a proposed scheme for the latter, according to the article reported by Xinlei Jia et al. [7], it speculates that the impurity electrons in N-type doping of the poly-plug will increase the risk of programming disturbance.

## 6. Conclusions

Multi-deck stacking is an effective way to increase the density of 3D NAND flash, while it can also reduce the difficulty of the channel etching process. This article mainly focuses on the impact of connection schemes in the dual-deck structure on device reliability. In the dual-deck structure without a poly-plug connection, the alignment issues between the upper and lower decks could lead to a decrease in the breakdown and data retention characteristics in the top cells of the lower deck. This is mainly due to the overlap mismatch of decks, which causes secondary etching for the top cells of the lower deck, resulting in a change in cell structure and local electric field enhancement effects. Meanwhile, an abnormal current drop phenomenon was observed in the dual-deck structure with the poly-plug connection scheme. The plug region becomes the channel current bottleneck due to the shielding effect on near WLs. In addition, the different structures of the poly-plug caused different shielding effects, resulting in the channel current dropping variation increasing. Therefore, for the structure without a poly-plug scheme, the article proposes an increase in the thickness of the oxide between the upper and lower decks and setting the top cells of the lower deck as virtual cells to avoid device breakdown and data retention issues. At the same time, for the structure with a poly-plug scheme, it proposes use of poly-plug N-type doping to avoid abnormal channel current dropping caused by inversion issues.

## Figures and Tables

**Table 1:** Comparison of the connection schemes between deck in 3D dual-deck NAND flash.

Connection Schemes between Decks	Without Poly-Plug	With Poly-Plug
Process	Gate stacking and channel poly-silicon one-time deposition	Gate stacking and channel poly-silicon deposition in two steps
Cost	Lower	Higher
Reliability issues	Degradation of the gate to channel breakdown and data retention characteristics of top cells in lower deck	Channel current dropping and variation increasing
The main mechanism of reliability degradation	Electric field enhancement effect at the location of abnormal structures	Electric field shielding effect on the nearby WLs due to the Poly-plug
Proposal schemes	1. Increasing oxide thickness between decks 2. Setting top cells in lower deck as Virtual cells	Poly-plug N-type doping
Schemes Impact	Storage capacity reducing	Program disturbance risk increasing [7]

## Data Availability

Data is unavailable due to privacy.
